# Rural-urban differences of neonatal mortality in a poorly developed province of China

**DOI:** 10.1186/1471-2458-11-477

**Published:** 2011-06-18

**Authors:** Bin Yi, Li Wu, Hong Liu, Weimin Fang, Yang Hu, Youjie Wang

**Affiliations:** 1 Department of Maternal and Child Health, School of Public Health, Tongji Medical College, Huazhong University of Science & Technology, Hangkong Road 13, Wuhan, 430030, China; 2 Department of Neonatology, Maternal and Child Hospital of Gansu Province, Qilihe North Street 143, Lanzhou, 730050, China

## Abstract

**Background:**

The influence of rural-urban disparities in children's health on neonatal death in disadvantaged areas of China is poorly understood. In this study of rural and urban populations in Gansu province, a disadvantaged province of China, we describe the characteristics and mortality of newborn infants and evaluated rural-urban differences of neonatal death.

**Methods:**

We analyzed all neonatal deaths in the data from the Surveillance System of Child Death in Gansu Province, China from 2004 to 2009. We calculated all-cause neonatal mortality rates (NMR) and cause-specific death rates for infants born to rural or urban mothers during 2004-09. Rural-urban classifications were determined based on the residence registry system of China. Chi-square tests were used to compare differences of infant characteristics and cause-specific deaths by rural-urban maternal residence.

**Results:**

Overall, NMR fell in both rural and urban populations during 2004-09. Average NMR for rural and urban populations was 17.8 and 7.5 per 1000 live births, respectively. For both rural and urban newborn infants, the four leading causes of death were birth asphyxia, preterm or low birth weight, congenital malformation, and pneumonia. Each cause-specific death rate was higher in rural infants than in urban infants. More rural than urban neonates died out of hospital or did not receive medical care before death.

**Conclusions:**

Neonatal mortality declined dramatically both in urban and rural groups in Gansu province during 2004-09. However, profound disparities persisted between rural and urban populations. Strategies that address inequalities of accessibility and quality of health care are necessary to improve neonatal health in rural settings in China.

## Background

China is the largest agricultural country in the world, with > 60% of its population living in rural areas. Compared with urban populations, Chinese rural populations have shorter life expectancy and disproportionally higher rates of overall mortality associated with a broad range of health problems [1,2]. In the past few decades, concerted efforts have been made to improve the health of rural populations. There has been great improvement of rural infant and child health. For example, infant mortality rates among rural populations have dropped from 37.0 to 18.4 per 1000 live births during the period from 2000 to 2008. However, rural-urban disparities in infant mortality have remained unchanged or even increased. For example, in 2000 and 2008 rural infant mortality rate was 2.5 and 2.8 times higher than urban infant rate [3]. Such large disparities of infant mortality rates are in contrast to the goals of Health China by 2020, which wants to narrow the rural-urban gap in health inequalities.

Although rural-urban disparities in neonatal mortality rates (NMR) have been consistently documented in China, these disparities are often overlooked [4,5]. Compared with the most advanced urban or developed coastal areas in China, NMR in remote and poor rural areas is unacceptably high. In 1999, NMR was 45.9 per 1000 live births in less-developed rural areas in Anhui province in China, while at same time NMR in Shanghai was 3.5 per 1000 live births [6,7].

Understanding the difference in neonatal deaths between rural and urban populations is important for assessing health needs of the populations and addressing health disparities. However, disparities in neonatal survival were less reported than those in infants and children aged < 5 years. To our knowledge, this is the first study, using data from Gansu province, to investigate disparities of neonatal health between rural and urban populations in China. Gansu province is located in north-west China, with a population of about 26 million at the end of 2008. It is one of the least developed provinces among the 27 provinces in mainland China by gross domestic product per capita, and had the largest gap in income between rural and urban populations in 2007 [8].

The objective of this study was to identify rural-urban disparities of NMR in Gansu province and to explore risk factors for neonatal death among rural populations.

## Methods

### Source of data

We used data from the Surveillance System of Child Death for Gansu Province so as to avoid misleading information deriving from relatively incomplete provincial death registry. A total of 12 counties and 4 districts were selected randomly from 78 counties and 19 districts to represent rural and urban surveillance units, respectively. The Surveillance System of Child Death in Gansu province covered about 3% of its 17.8 million rural population and 19% of its 8.4 million urban populations. Within the surveillance system, infant deaths occurring in hospitals were reported via computer network to the Maternal and Children's Hospital of Gansu Province. For deaths arising elsewhere, one trained health worker was assigned to each community or village and was responsible for registering the deaths. Every child death is required by law to be reported by the parents. Data from the surveillance system were verified twice yearly by provincial supervisors through sampling survey. Thus information from the surveillance system is regarded as having high reliability. The period 2004 to 2009 was the most recent continuous period for which complete data were available.

Data on sex, birth date, birth weight, gestational age, death date, death cause, place of death, hospitalization or not, with or without treatment before death, and criteria of diagnosis were extracted from children's death records in the Surveillance System of Child Death for analysis in this study.

### Variable definition

Information on direct cause of death in neonates derived from hospital records for deaths occurring in hospital and from pediatrician panel opinions based on information provided by parents or doctors for those occurring elsewhere. All death causes were initially classified into 35 categories and coded using ICD-10 by pediatricians and epidemiologists working for the surveillance system. In this study, we combined death causes except birth asphyxia, preterm or low birth weight, congenital malformation, and pneumonia as "others" for analysis. Birth asphyxia, preterm or low birth weight, congenital malformation, and pneumonia comprised 86.4% (n = 677) of rural neonatal deaths and 91.5% (n = 586) of urban neonatal deaths.

Births were classified as either "rural" or "urban" on the basis of the mother's residence. Urban/rural residence was determined on the basis of the code developed by National Bureau of Statistics of China in regard to statistical divisions of urban and rural areas. In this study, areas coded 100-123 were grouped into urban residence and 200-220 into rural residence.

### Data analysis

Statistical analysis was performed using statistical software SPSS version 11. Neonatal, late neonatal, and infant mortality rates and cause-specific NMR were calculated per 1000 live births. Rate ratios of death for rural neonates compared with urban neonates were calculated with 95% confidence intervals (CIs). Differences between groups were assessed by Chi-square test

Ethics approval for this research was obtained from the Research Ethics Committee of Tongji Medical College, Huazhong University of Science and Technology.

## Results

Between 2004 and 2009 there were 115,765 live births and 1263 neonatal deaths in the surveillance sites of Gansu province. NMR declined in rural and urban populations over the period, with average yearly declines of 5.8% and 8.0% for rural and urban NMR, respectively, during 2004-09. Overall, rural and urban NMR declined by 34.8% (from 20.9 to 13.6 per 1000 live births) and 47.7% (from 9.5 to 5.0 per 1000 live births), respectively. Rural areas consistently had higher NMR than urban areas (Table 1). Average NMR during the period was significantly higher in rural neonates (17.8 per 1000 live births) than in urban neonates (7.5 per 1000 live births) (*P *< 0.01), representing a 2.4-fold difference. Births to rural mothers accounted for 32.9% of all births in the surveillance areas and 53.6% of all deaths, suggesting disproportionately higher mortality in rural births.

**Table 1:** Neonatal mortality rate (per 1000 live births) by rural-urban residence in Gansu province, China, 2004-09

Year	Urban			Rural			Rural-urban Rate Ratio(95%CI)
		
	Live birth	Neonatal death	Mortality rate(95%CI)	Live birth	Neonatal death	Mortality rate(95%CI)	
2004	11765	112	9.5(7.8-11.3)	5705	119	20.9(17.2-24.6)	2.2(1.7-2.8)
2005	11017	80	7.3(5.7-8.8)	5544	132	23.8(19.8-27.8)	3.3(2.5-4.3)
2006	12776	100	7.8(6.4-9.3)	6200	133	21.4(19.1-23.8)	2.7(2.0-3.4)
2007	14606	115	7.9(6.4-9.3)	6588	102	15.5(13.5-17.5)	2.0(1.5-2.5)
2008	13888	111	8.0(6.5-9.5)	6961	95	13.6(10.9-16.4)	1.7(1.3-2.0)
2009	13663	68	5.0(3.8-6.2)	7052	96	13.6(10.9-16.3)	2.7(2.0-7.6)
2004-2009	77715	586	7.5(6.9-8.1)	38050	677	17.8(16.5-19.1)	2.4(2.1-6.2)

The leading causes of neonatal death for both rural and urban neonates were birth asphyxia, preterm birth or low birth weight, congenital malformation, and pneumonia. NMR for each cause was significantly higher in rural versus urban neonates (Table 2). The rural-urban NMR ratios ranged between 1.54 for congenital malformation and 2.54 for preterm or low birth weight (Table 2).

**Table 2:** The cause specific neonatal mortality rate (per 1000 live births) by rural-urban residence in Gansu province, 2004-09

Death cause	Urban			Rural			Rural-urban rate ratio(95%CI)
		
	Neonatal death	Mortality rate(95%CI)	Proportion(%)	Neonatal death	Mortality rate(95%CI)	Proportion(%)	
Birth asphyxia	211	2.7(2.4-3.1)	36	233	6.1(5.3-6.9)	34.4	2.2(1.9-4.6)
Preterm or low-birth weight	150	1.9(1.6-2.2)	25.6	187	4.9(4.2-5.6)	27.6	2.5 (2.1-6.8)
Congenital malformation	110	1.4(1.2-1.7)	18.8	83	2.2(1.7-2.6)	12.3	1.5 (1.2-1.6)
Pneumonia	65	0.8(0.7-1.1)	11.1	82	2.2(1.7-2.6)	12.1	2.6(1.8-4.8)
Others	50	0.6(0.5-0.9)	8.5	92	2.4(1.9-2.9)	13.6	3.8(2.6-27.8)
Total	586	7.5(6.9-8.1)	100	677	17.8(16.5-19.1)	100	2.4 (2.1-6.2)

Among rural neonatal deaths, 57.0% were boys (Table 3). For rural boys and girls NMR was 18.9 and 16.5 per 1000 live births, respectively. Among urban neonatal deaths, 60.2% were boys (Table 3). For urban boys and girls NMR was 8.6 and 6.4 per 1000 live births, respectively. Male-to-female ratio in neonatal mortality in urban and rural areas was 1.34 and 1.14, respectively.

**Table 3:** The Characteristics of rural and urban neonatal mortality of Gansu province, 2004-09

Characteristics	Urban N (%)	Rural N (%)	χ^2^	P
**Sex**				
Male	353 (60.2)	386(57.0)	1.34	0.25
Female	233(39.8)	291(43.0)		
**Gestational age(week)**				
Very premature, < 33	90 (15.4)	106 (15.6)	19.73	< 0.001
Moderate premature,33-37	157 (26.8)	117 (17.3)		
Term or postterm, ≥ 38	339 (57.8)	454 (67.1)		
**Birth weight(g)**				
Very low < 1500	49 (8.4)	24 (3.6)	18.66	< 0.001
Moderately low,1500-2499	171 (29.2)	182 (26.9)		
Normal, 2500-3999	358 (61.1)	458 (67.6)		
≥ 4000	8 (1.4)	13 (1.9)		
**Medical care before death**				
Hospital care	529 (90.3)	433 (64.0)	131.85	< 0.001
Outpatient care	11(1.9)	126 (18.6)		
No medical care	46 (7.8)	118 (17.4)		
**Death season**				
Spring	142(24.2)	152(22.4)	2.38	0.5
Summer	145(24.7)	152(22.4)		
Autumn	160(27.3)	192(28.4)		
Winter	139(23.7)	181(26.7)		
**Death place**				
Hospital	491 (83.8)	424 (62.6)	70.72	< .001
Way to hospital	11 (1.9)	24 (3.6)		
Home	84(14.3)	229 (33.8)		

A higher proportion of rural neonatal death (67.6%) was within normal gestational age and birth weight compared with urban areas (61.1%; P < 0.01) (Table 3). More than one third (33.8%) of rural neonatal deaths took place at home, while 14.3% of urban neonatal death took place at home. There were significantly higher rural neonatal deaths (37.4%) than urban neonatal deaths (16.2%) that took place out of hospital, and 17.4% rural versus 7.8% urban neonatal deaths occurred without receiving medical care before death. Located in northwest China, Gansu province has average winter temperature < 0°C or far below 0°C in some areas, but this study did not show any difference of rural and urban death distribution by season (Table 3).

As shown in Table 4 early neonatal, neonatal, and postnatal death and infant death in rural areas presents a different pattern from that in urban areas. Among rural and urban deaths of children aged < 5 years neonatal death accounted for 59.0% and 67.6%, respectively, while for infants the proportion was 93.3% and 98.1%, respectively. Most neonatal deaths took place in the first 7 days after birth, accounting for 80.9% and 87.2% of rural and urban neonatal deaths, respectively. Neonatal deaths taking place on the first day of life accounted for 47.3% and 42.3% of rural and urban neonatal deaths, respectively.

**Table 4:** Relationship between mortalities by rural-urban residence of Gansu province, 2004-09

Residence	Early neonatal death	Neonatal death	Infant death	Under-five death	Neonatal death as of% under-five Death	Neonatal death as of% infant Death	Early neonatal death as of % neonatal death
Rural	549	677	866	964	70.2	78.2	81.1
Urban	501	586	671	760	77.1	87.3	85.5

## Discussion

Neonatal health is among the most striking examples of health disparity in the world. Most of the 4 million neonatal deaths taking place each year occur in the most deprived countries [9]. The disparity of NMR between rich and poor countries is unacceptably large and continues to increase [10].

The present study identified considerable differences in neonatal deaths including direct causes of death between rural and urban populations in the poorly developed province of Gansu, China. We found that although there was a significant decline in NMR in rural areas during recent years that for all causes and leading causes of death, rural NMR was much higher than urban NMR. Most previous reports revealed that distribution of causes of neonatal deaths varied between lower and higher neonatal mortality settings [11-13]. In this study, we found that the leading causes of neonatal death for both rural and urban populations were the same, including birth asphyxia, preterm birth or low birth weight, congenital malformation, and pneumonia, accounting for 91.6% and 84.6% of urban and rural neonatal deaths, respectively. In general, except for congenital malformation, most neonatal deaths caused by preventable or treatable conditions can be avoided if appropriate measures are taken. The risk of death due to birth asphyxia accounted for more than one-third in both rural and urban populations. Compared with the data from other regions in China, the proportion of death due to birth asphyxia was higher in Gansu province.

Several factors might contribute to the higher risk of neonatal death for rural babies in Gansu. Socioeconomic difference is likely the main underlying cause of the disparity. Although it is thought that social and economic determinants of health are important for improvement of child survival, they are not well understood. In this study, based on data obtained from Gansu province statistics department, Gansu province has the greatest inequity of GDP per capita between rural and urban populations in China, with an urban to rural income ratio of 4.5 in 2007 [14]. This inequity may impact neonatal death in several ways including lower education of mother, poor nutrition for mother and fetus, reduced access to prenatal health service, and reduced quality of medical care, especially the neonatal intensive medical care. We observed a greater percentage of rural versus urban neonatal deaths taking place out of hospital and without medical care before death. A higher proportion of rural compared with urban death from birth asphyxia and pneumonia arising out of hospital suggests delayed presentation, diagnosis, or treatment. Most deaths due to birth asphyxia and pneumonia are avoidable once treatment is given. In this study birth asphyxia caused death among 55.6% in rural areas and 7.2% in urban areas, and among neonatal death due to pneumonia 39.3% rural and 2.3% urban neonates did not received any medical care before death. The most common reason for not receiving medical care was health facilities beyond reach; the second was lack of money (data not shown).

Reducing NMR is crucial to reduce infant mortality rate and mortality rate in children aged < 5 years. Neonatal death accounted for 59.0% and 67.6%, respectively, of death occurring in rural and urban children aged < 5 years and 80.6% and 89.4%, respectively, of that in rural and urban infants. Therefore, further reduction of child mortality will depend on substantial improvement of neonatal and early neonatal survival

We found that in urban and rural areas neonatal death rate for boys versus girls was 1.41 and 1.02 times higher, respectively. This finding is in contrast to most previous reports [15,16]. In 2000, infant mortality was 33.7 per 1000 live births for girls compared with 23.9 per 1000 for boys [17]. Preference for sons leads to excess female infant and neonatal mortality in China, especially in rural areas. It was estimated that male-to-female ratios were 1.3 for neonatal mortality [18]. It is well known that girls have biological survival advantage in neonatal and infant periods [19]. Our findings suggest that sex discrimination hardly exists in urban population, but might exist in rural population.

Our research was subject to several limitations. We analyzed data from only one province, Gansu, and our results may not be nationally representative. However, Gansu is the province with the largest economic gap between rural-urban residences in China. Our results are likely to reflect the situation of neonatal death in disadvantaged provinces or areas of China. Second, information from of this study was from the Surveillance System of Child Death of Gansu, which does not contain data on such factors as prenatal care, mother's age, education, lifestyle, and environment. This limitation restricted us to analyze the underlying causes of higher neonatal death in rural population.

Neonatal death is associated with events surrounding delivery, pregnancy, and neonatal care following birth, and closely linked to the place of delivery and care provided at delivery and quality of in-hospital medical care. The high proportion of birth asphyxia in our study suggests that adequate resuscitation of newborns is of extreme importance to save the life of baby.

Understanding differences of neonatal deaths between rural and urban population is important for assessing the health needs of the population and addressing health disparities and for formulating effective strategies to improve the health of rural infants. One strategy could be increasing institutional delivery rates, based on the finding that nearly half neonatal death occurred during the first day of life, and medical treatment can be effective. It is reported that 30% of rural deliveries occurred out of hospital in Gansu province in 2008. Another strategy could focus on improving intensive care in hospital.

NMR in Chinese urban populations is relatively low and close to the level of developed countries, even in the less developed urban areas such as those in Gansu Province. Thus overcoming inequities that exist between rural and urban newborns is essential to reach China's millennium development goal 4 and improve overall health of children.

## Conclusion

In Gansu province, rural NMR was worse than urban NMR, although NMR declined in urban and rural groups during 2004-09, profound disparities of neonatal deaths persisted between rural and urban populations.

## Competing interests

The authors declare that they have no competing interests.

## Authors' contributions

YW conceived the idea and designed the study, LW, HL, MF and YH analyzed the data. YW and BY drafted the manuscript. All authors contributed to the interpretation of results and approved the final manuscript.

## Pre-publication history

The pre-publication history for this paper can be accessed here:

http://www.biomedcentral.com/1471-2458/11/477/prepub
